# Robotically facilitated virtual rehabilitation of arm transport integrated with finger movement in persons with hemiparesis

**DOI:** 10.1186/1743-0003-8-27

**Published:** 2011-05-16

**Authors:** Alma S Merians, Gerard G Fluet, Qinyin Qiu, Soha Saleh, Ian Lafond, Amy Davidow, Sergei V Adamovich

**Affiliations:** 1 Department of Rehabilitation and Movement Sciences, University of Medicine and Dentistry of New Jersey, Newark, NJ; 2 Department of Quantitative Methods, University of Medicine and Dentistry of New Jersey, Newark, NJ; 3 Department of Biomedical Engineering, New Jersey Institute of Technology, Newark, NJ

## Abstract

**Background:**

Recovery of upper extremity function is particularly recalcitrant to successful rehabilitation. Robotic-assisted arm training devices integrated with virtual targets or complex virtual reality gaming simulations are being developed to deal with this problem. Neural control mechanisms indicate that reaching and hand-object manipulation are interdependent, suggesting that training on tasks requiring coordinated effort of both the upper arm and hand may be a more effective method for improving recovery of real world function. However, most robotic therapies have focused on training the proximal, rather than distal effectors of the upper extremity. This paper describes the effects of robotically-assisted, integrated upper extremity training.

**Methods:**

Twelve subjects post-stroke were trained for eight days on four upper extremity gaming simulations using adaptive robots during 2-3 hour sessions.

**Results:**

The subjects demonstrated improved proximal stability, smoothness and efficiency of the movement path. This was in concert with improvement in the distal kinematic measures of finger individuation and improved speed. Importantly, these changes were accompanied by a robust 16-second decrease in overall time in the Wolf Motor Function Test and a 24-second decrease in the Jebsen Test of Hand Function.

**Conclusions:**

Complex gaming simulations interfaced with adaptive robots requiring integrated control of shoulder, elbow, forearm, wrist and finger movements appear to have a substantial effect on improving hemiparetic hand function. We believe that the magnitude of the changes and the stability of the patient's function prior to training, along with maintenance of several aspects of the gains demonstrated at retention make a compelling argument for this approach to training.

## Background

Sensorimotor impairments and participation restrictions remain a pervasive problem for patients post stroke, with recovery of upper extremity function particularly recalcitrant to intervention. 80% to 95% of persons demonstrate residual upper extremity impairments lasting beyond six months after their strokes [1]. One of the issues that may contribute to less than satisfactory outcomes for the upper extremity is the complexity of sensory processing and motor output involved in normal hand function. There is a vital need to develop rehabilitative training strategies that will improve functional outcomes and real-world use of the arm and hand. In an attempt to address this need, many researchers are developing robotic-assisted arm training devices in concert with strategically placed virtual targets or complex virtual reality gaming simulations. Integrated whole arm activities are difficult because most robotic devices are designed for upper arm motion and not for grasp and fine motor activities. An additional hurdle stems from multiple lines of inquiry in animal and human motor learning and neuroplasticity literature, that indicate that sufficient task complexity seems to be a factor in upper extremity motor skill development and cortical plasticity [2-5], requiring more complex training activities than those typically seen in the robotic rehabilitation literature.

In an effort to improve upper extremity outcomes post-stroke we have concentrated on hand training. Our past work has used virtual reality gaming simulations to exercise finger movements of a stationary hand, including functional individual finger motions and whole hand opening/closing, to interact with simple interactive virtual environments. Subjects showed improvement in the kinematics of the movements as well as in dexterity as measured by clinical tests of hand function [6-8]. This intervention utilized current neurophysiological findings regarding the importance of repetitive, frequent and intensive practice for skill development and motor recovery [9-13].

As we do not know the best training strategy to facilitate recovery of hand function and recognizing the neural interaction of arm and hand and the importance of training using functionally complex movements, we asked the question whether training the arm and hand in an integrated manner would promote better motor recovery outcomes than previously reported hand-only training. In this paper we describe a study that used interactive gaming simulations interfaced with adaptive robots to provide a multi-faceted environment to test the assumption that training the entire upper extremity, including fingers, as a unit will improve the hemiparetic hand of patients post-stroke and importantly that the kinematic changes gained through this type of practice would transfer to untrained real world arm/hand activities.

## Methods

### System

#### Hardware

All simulations in this study utilized CyberGlove^© ^(Immersion) instrumented gloves for hand tracking. A CyberGrasp^© ^(Immersion), a lightweight, force-reflecting exoskeleton that fits over the CyberGlove was used to facilitate individual finger movement in patients with more pronounced deficits. Two of the four simulations use the Flock of Birds (Ascension Technologies) motion sensors for arm tracking and the other two use the Haptic Master robot (Moog FCS Corporation). Please see [14,15] for full description of the hardware.

#### Simulations

**Figure 1:**
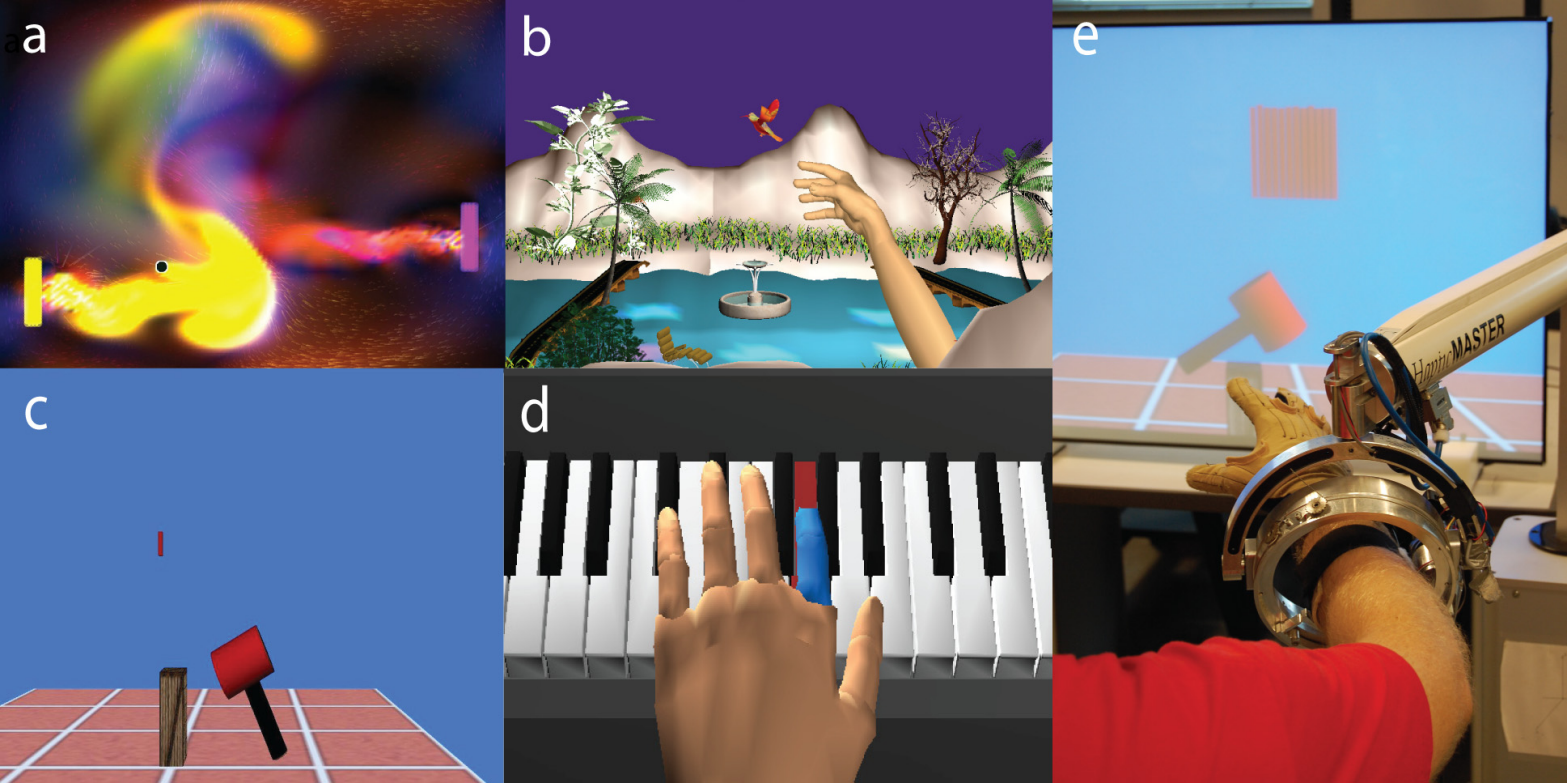
**Simulations**. Screen shots for simulations utilized during this training study *a*. Plasma Pong, *b*. Hummingbird Hunt, *c*. Hammer Task, *d*. Virtual Piano. *e*. Training setup.

Four gaming simulations were developed. All four simulations integrate components of upper arm movement with wrist and hand movement. Plasma Pong^© ^(Steve Taylor, 2007) was adopted from an existing game in which the game control was transferred from the computer mouse to the CyberGlove. In this game (Figure 1a), the pong paddle is moved vertically using shoulder flexion/extension while the moving ball is engaged horizontally, using rapid finger extension. The Hummingbird Hunt simulation depicts a hummingbird moving through an environment filled with trees, flowers and a river (Figure 1b), providing practice in the composite movement of arm transport, hand-shaping and grasp. A pincer grip is used to catch and release the bird while it is positioned in different locations of a 3D workspace. The Hammer Task (Figure 1c) trains a combination of three dimensional reaching and repetitive finger flexion/extension. The subjects reach toward a virtual wooden cylinder, stabilize their upper arm and then use either finger extension or flexion to hammer the cylinders into the floor. The Virtual Piano simulation consists of a complete virtual piano (Figure 1d) that plays the appropriate notes as they are pressed by the virtual fingers using the CyberGlove with or without the CyberGrasp. Please see [7,16] for full description of the simulations. Figure 1e shows the experimental set-up for the integration of the Haptic Master robot, the arm supporting gimbal and the CyberGlove.

### Subjects

Twelve subjects (8 male, 4 female), mean (SD) age of 55 (14) years, and mean (SD) time post stroke of 5 (5) years, (range 9 months to 15 years) participated in this study. Inclusion criteria were subjects at least 6 months post-stroke, wrist extension of at least 10°, finger extension of 10° and not receiving any other therapy at the time of the study. Exclusion criteria included severe aphasia, hemispatial neglect and botulinum toxin injections within the past 3 months. The Chedoke McMaster Arm (CMA) and Hand (CMH) Impairment Inventories [17] and a composite of upper extremity Ashworth scores were used to categorize the impairment levels of the subjects (see Table 1 for demographic and impairment data). Consent was obtained from all subjects and the Internal Review Boards of both universities approved the protocol. Subjects trained on all four simulations during 2-3 hour sessions for eight days. Training was divided equally between the four simulations. Total training time started on day one at two hours and increased in fifteen-minute increments during Week 1. Training time started and remained at three hours on all four days of Week 2.

**Table 1:** Subject characteristics

Subject	Age	Years Post CVA	Gender	CMA	CMH	Ashworth^a^
S1	63	3 yrs	Male	6	5	3
S2	53	10 mo	Female	7	4	5
S3	68	15 yrs	Male	4	3	7
S4	54	2 yrs	Male	6	4	3
S5	70	8 yrs	Female	7	5	1
S6	72	12 yrs	Male	5	4	6
S7	61	4.5 yrs	Female	5	5	4
S8	62	1.5 yrs	Male	6	6	3
S9	25	9 mo	Male	5	4	5
S10	47	9.5 yrs	Male	4	3	6
S11	38	3 yrs	Female	6	6	3
S12	54	11 mo	Male	7	6	0

Abbreviations: CMA, Chedoke McMaster Arm Stage; CMH, Chedoke McMaster Hand Stage; yrs, years; mo, months.

^a^Ashworth denotes Composite of Ashworth Grades for Shoulder Extensors, Elbow Flexors and Wrist Flexors.

### Measurement

Two timed clinical tests served as our primary outcome measures: Jebsen Test of Hand Function (JTHF) and Wolf Motor Function Test (WMFT) [18,19]. Both the impaired and unimpaired arm/hand were tested for each clinical test. For the WMFT 120 seconds were recorded when the subject could not perform the subtest [20], while for the JTHF we used 45 sec as a score for a failed subtest. Similar to other reported studies, we eliminated the writing component of the JTHF [6,21]. In each session, the JTHF was administered three times and the mean of the three scores was used for analysis. Stroke subjects were tested prior to training, immediately post training and at least three months after training. Subjects were at least 6 months post-stroke and reported to be neurologically stable. To confirm the stability of their motor function and absence of confounding spontaneous recovery, for each clinical test, we conducted two baseline tests on a subset (N = 8), of the twelve subjects with stroke, two weeks before and one day before the onset of training. In addition, seven age-matched, neurologically healthy subjects performed the JTHF, three times, at two- week intervals, three times per session.

The secondary measures were the kinematic measures obtained from the Hammer task and the Virtual Piano. We have designed the simulation tasks to have both discrete and continuous movements. The Virtual Piano and the Hammer Task consist of discrete movements with a definite beginning and end, making them more amenable to kinematic analyses. For the Hammer task, these included, hand-path length, maximal extension of the Metacarpal-phalangeal joints (MPJ), time to complete the task (duration) which includes the reaching and hammering phase for each cylinder, the smoothness of the hand trajectory and the deviation of the wrist position in 3D space during hammering [22]. Smoothness of the trajectories was evaluated by integrating the third derivative of the trajectory length. This numerically describes the ability to produce smooth, coordinated, reaching movements [14,23]. Hand deviation was measured as the mean distance of the hand from the target during hammering (using finger flexion and extension) and is considered a measure of proximal stability and shoulder stabilization during hand-object interaction [22]. For the Virtual Piano, kinematic measures included accuracy, measured by the percent of correct key presses, time to complete the task (duration), which includes both hand transport and key press time for each note in the song, and fractionation, the ability to isolate the movement of each finger, measured as the difference in MCP joint angle between the cued finger and the most flexed non-cued finger.

### Data Analysis

The subjects were evaluated three times on the primary outcome measures, with two pre-planned contrasts: Pre-test minus Post-test, and Pre-test minus Retention-test. Data sets for pre-test, post-test and retention were each evaluated for normality using the Kolmogorov-Smirnov Test. While JTHF scores were normally distributed (p > 0.20), scores for the WMFT were positively skewed (p < 0.1) because of two of the most involved subjects. We have performed all statistical tests using clinical scores of all 12 subjects, as well as of 10 subjects (with the two most involved subjects removed), with similar results. Therefore, we will report the outcomes of parametric statistical tests on all 12 subjects. At the same time, the Pre_minus_Post and Pre_ minus_Retention differences in the WMFT and JTHF clinical scores of the 12 subjects were normally distributed (p > 0.2). Therefore, we will use these data to compare the mean percent improvement between the Pre-test and Post-test scores demonstrated by subjects in this study with those in our previous studies (see Discussion).

For the clinical measures, first, the combined scores of the two tests (WMFT, JTHF) were subjected to a repeated measures ANOVA with factors Test (JTHF, WMFT) and Measurement Time (Pre-test, Post-test, Retention). The Pre-test score was calculated as an average of the two baseline scores for subjects with two pre-training measurements obtained two weeks and one day before the training. Preplanned post-hoc comparisons, Pre-test versus Post-test and Pre-test versus Retention were made for the combined clinical test using two separate, repeated measures ANOVAs with repeated measures of Test (JTHF, WMFT) and Measurement Time (Pre-test, Post-test) or Measurement Time (Pre-test, Retention). The degrees of freedom for all ANOVA tests were adjusted using Greenhouse-Geisser corrections. Finally, preplanned post-hoc comparisons, Pre-test versus Post-test and Pre-test versus Retention were made using two separate, repeated measures ANOVAs for each of the two tests. Eta-squared statistics were used to calculate estimates of effect sizes for group comparisons.

All the kinematic measurements described above were normally distributed. To derive a start measure (SM), performance scores were pooled over the first two days of therapy in order to enhance data stability and reduce potential effects due to subjects acclimating to the robotic system and the virtual environments on Day 1. Performance scores from the last two days were also pooled to obtain a larger data sample for enhanced data stability of the end measure (EM) [6,24]. For the Hammer Task four separate repeated measures ANOVAs with factor, Measurement Time (SM, EM) were used to evaluate changes in arm kinematics (Duration, Hand Path Length, Smoothness and Hand Deviation). For the Piano task, three separate repeated measures ANOVAs with factor, Measurement Time (SM, EM) were used to evaluate changes in hand kinematics (Fractionation, Duration, Accuracy).

The percent change in the mean clinical scores was calculated as 100 multiplied by the difference between Pre-test and Post-test mean scores, divided by Pre-test mean score. This allowed for a comparison with the outcomes of a former study where we used the previous version of our VR training system [6]. For kinematic measures, the percent changes were calculated in similar fashion using starting measure SM and end measure EM as described above.

## Results

### Kinematic Analyses

**Figure 2:**
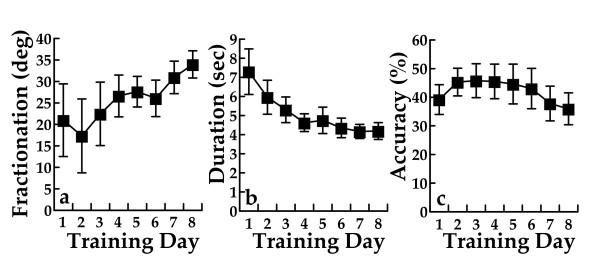
**Piano trainer kinematic analyses**. *a*. Daily averages during Virtual Piano training for finger fractionation defined as the difference between the angle of the MCP joint of the cued finger and of the most flexed non-cued finger. Higher scores indicate better performance. Averages for 10 subjects are shown (two subjects who used the CyberGrasp haptic device during virtual piano training are not included in this analysis). *2b*. Daily averages for all 12 subjects in the time to press each key during piano training. *2c*.Daily averages of number of correct keys pressed divided by total keys pressed for all 12 subjects. Error bars = Standard Error of the Mean.

Figure 2 displays the group average daily change in the Piano task for finger fractionation (2a), average movement duration for each note in a song (2b), accuracy of key presses (2c). Two subjects needed to use haptic assistance from the CyberGrasp for this activity and were therefore eliminated from the group calculations for fractionation (ability to isolate their finger movement). As a group the other ten subjects significantly improved in fractionation (Table 2) showing a 39% change. There was a significant improvement in the time to complete the task showing a 19% change without a subsequent change in accuracy (Table 2), indicating that the subjects were able to do the task faster without a substantive change in accuracy. This is thought to be consistent with motor learning [25].

**Table 2:** Kinematic variables

	Pre-Test	Post-Test	F	P
Virtual Piano Trainer				
Finger Fractionation (deg)^a^	23.3 (18.8)	33.0 (10.2)	5.7	0.044
Time to Press Each Key (sec)	5.82 (2.4)	4.72 (1.6)	5.4	0.04
Accuracy^a^	0.44 (0.17)	0.40 (0.23)	0.54	0.48
				
Hammer Task				
Time per Cylinder (sec)	31 (19)	15 (7)	13.6	0.005
Arm Endpoint Path Length (m)	1.2 (.62)	0.72 (.23)	14.7	0.003
Arm Endpoint Smoothness, *10^3^	62.03 (86.7)	15.1(16.4)	5.2	0.05
Arm Endpoint Deviation	87 (50)	42 (19)	19.2	0.002
Peak MPJ Extension	22.5 (16)	19.5 (19)	2.42	0.16

Abbreviations: MPJ, Metacarpal-phalangeal joint.

^a^For all measures except finger fractionation and accuracy, lower scores indicate better performance.

^b^Mean (standard deviation)

**Figure 3:**
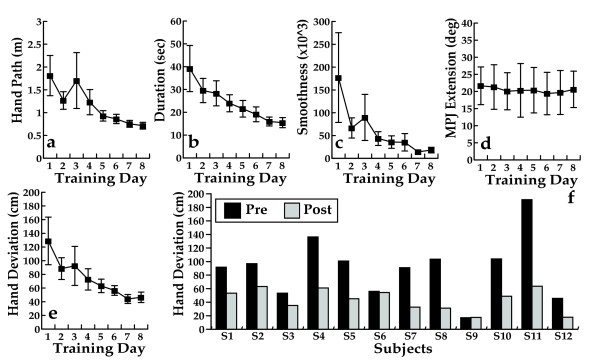
**Hammer simulation kinematic analyses**. Daily average for all twelve subjects during Hammer Task training in *a*. the length of the path required to complete ten targets. *b*. time required to hammer each virtual cylinder *c*. in hand trajectory smoothness quantified as normalized integrated jerk (values are dimensionless, lower scores indicate smoother path with fewer subunits). *d*. peak finger extension. *3e*. hand deviation calculated as the cumulative excursion of the hand position in 3D space from the center of the target starting at the time target is acquired until completion of hammering (lower scores indicate more stability). *3f*. Individual subjects start measure (average of first two training days), and end measure (average of last two training days) for all twelve subjects in average hand deviation during hammer task training. Error bars = Standard Error of the Mean.

Figure 3 summarizes group changes during the Hammer task in the hand path length (3a), duration (3b), smoothness of the arm trajectories (3c), peak MPJ extension (3d), group changes in hand deviation (3e) and individual subject improvement in hand deviation (3f). There was a significant decrease in four of the five kinematic variables (Table 2). The time needed to complete each hammering task decreased, showing a 47% change. The hand path decreased in length, by 41% and improved in smoothness by 76%. The improvement in movement time and path length appears to be related to changes in proximal segment function as finger extension (3d), did not change significantly. A decrease in end-point deviation is an indicator of proximal stability. As a group, the subjects improved the proximal stability of the arm while the fingers were repeatedly extending during the hammering task (Table 2), showing a 51% change. Figure 3f indicates that eleven of the twelve subjects improved in this measure with smaller bars indicating less superfluous proximal segment movement while distal segments interacted with the target. Lang cites the ability to maintain proximal segments stationary during distal task performance as an important construct in overall upper extremity functional ability [26].

### Clinical Analyses

First, we evaluated the effects of training on the combined clinical score of the two timed tests (WMFT, JTHF) that served as our primary outcome measures. The repeated measures ANOVA showed a significant effect of Measurement Time (F(2,22) = 13.2, G-G adjusted p = 0.002, partial eta-squared 0.55, observed power (at alpha = 0.05) equal to 0.99), with no significant Clinical Test × Measurement Time interaction. The subsequent separate ANOVAs with a repeated factor Measurement Time (Pre-test, Post-test, Retention) demonstrated statistically significant effects of training for each individual clinical test, WMFT (F(2,22) = 8.35, G-G adjusted p = 0.01, eta squared = 0.43, observed power 0.94) and JTHF (F(2,22) = 9.92, G-G adjusted p = 0.001, eta squared = 0.47, observed power 0.97). Finally, both pre-planned post hoc comparisons (Pre-test versus Post-test and Pre-test versus Retention) for each of the two individual clinical tests were also significant (Table 3). As a group, the 12 subjects showed a percent improvement from Pre-test to Post-test of 22% in the WMFT (eta squared = 0.83) and 20% in the JTHF (eta squared = 0.71).

**Table 3:** Training Effects for Clinical Tests

	Pre-test versus Post-test				Pre-test versus Retention			
Test	F_1,11_	P	ES^a^	Power^b^	F_1,11_	P	ES^a^	Power^b^
WMFT	54.8	0.00001	0.83	0.99	0.4	0.008	0.49	0.99
JTHF	27.0	0.0003	0.71	0.84	8.1	0.02	0.43	0.74

Abbreviations: ES, Effect Size, WMFT, Wolf Motor Function Test, JTHF, Jebsen Test of Hand Function

^a^Effect sizes were calculated as partial eta squared.

^b^Observed power at alpha = 0.05

In a separate analysis on a subset of eight subjects, we verified the absence of spontaneous recovery by conducting two baseline tests, two weeks before and one day before the beginning of the training. Scores for both WMFT and JTHF were normally distributed (Kolmogorov-Smirnov normality test, p > 0.10). A repeated measures ANOVA with factors Clinical Test (WMFT, JTHF) × Measurement Time (Pre-test 1, Pre-test 2, Post-test, Retention) showed a significant effect of Time (F(3,21) = 10.7; G-G adjusted p = 0.001). Pre-planned post-hoc tests (Pre-test 1 versus Pre-test 2 and Pre-test 2 versus Post-test) showed no difference between the Pre-test 1 and Pre-test 2 for the composite clinical test (F(1,7) = 0.73, p = 0.42) while the composite clinical score at Post-test was significantly better than at Pre-test 2 (F(1,7) = 12.75, p = 0.009). The interaction effect of Clinical Test × Measurement Time was non-significant. Separate repeated measures ANOVAs showed no significant difference in the baseline scores between Pre-test 1 and Pre-test 2 in any of the clinical tests. Mean (SD) scores for WMFT were equal to 53.6 (15.6) and 54.6 (11.0); and for JTHF were equal to 100.3 (38.8) and 103.4 (36.4), respectively. At the same time, Post-test mean (SD) scores were significantly better: 40.0 (8.4) for WMFT and 84.6 (39.0) for JTHF. These analyses indicate the stability of the subjects' motor function prior to training as evaluated by our two clinical tests.

### Interpretive measures of clinical outcomes

Six out of 12 subjects demonstrated a percent improvement in their WMFT scores after 8 days of intensive training larger than 30% (range: 30-41), while the other half demonstrated smaller but still substantial percent improvement (range: 10-24). The mean (95% CI) decrease of 16 (13-22) sec in the WMFT time substantially exceeds the reported group change of 2 seconds needed to be regarded as a clinically important difference on the WMFT [27]. To indicate a true change for an individual subject in the time to complete the WMFT, that is a change beyond possible measurement error, the difference in score of an individual subject has to reach 4.36 sec [27]. In this study each subject exceeded the minimum detectable change of 4.36 seconds (range 5.7 to 33.2 sec). Additionally, Wolf et al. [28] cite the completion of an item on a clinical test of upper extremity function at post-test, which a subject was unable to complete at pre-test, as a clinically significant change. One subject was unable to complete the checker task at pre-test but was able to do it at the retention test. This same subject was also unable to complete the picking up small objects and self feeding tasks of the JTHF at pre-test but did complete them at post-test and retention. It is interesting to note that these changes in hand dexterity were observed in both clinical tests.

**Figure 4:**
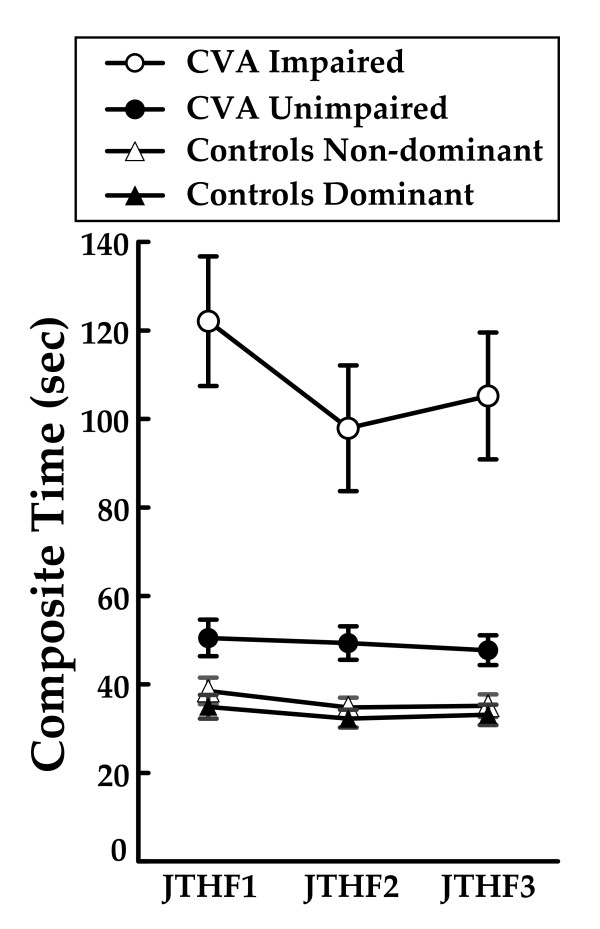
**Jebsen test of hand function comparison**. The composite time for the Jebsen Test of Hand Function at three testing points for the 12 subjects with strokes (JTHF1 = Pre-test, JTHF2 = Post-test, JTHF3 = Retention, Impaired Hand = open circles, Unimpaired Hand = solid circles), and the seven aged matched controls (Non-Dominant Hand = open triangles, Dominant hand = solid triangles). Error bars = Standard Error of the Mean.

To evaluate the functional relevance of the observed improvement in the JTHF scores, we compared the performance of the hemiparetic arm with that of the arm ipsilateral to the lesion, as well as with the scores of nine age-matched, neurologically healthy controls (Figure 4). All subjects were tested on three separate occasions, with two weeks between the tests. The control subjects were able to complete the six activities of the JTHF on average in 33 (95% CI: 29-38) sec using their dominant hand and in 36 (31-41) sec using their non-dominant hand. The subjects with stroke required 49 (41-57) sec to complete the six activities using their uninvolved hand and when using their impaired hand, improved from 122 (90-154) sec to 98 (66-129) sec after training. Measures for the uninvolved hand and the controls were stable across the three time frames with only the hemiparetic hand showing improved scores after training.

It is believed that patients in the chronic phase post-stroke, in general, are less physically active and do not receive physical or occupational therapy. Therefore, there is some concern that positive results of training studies are due mainly to the large increase of activity afforded by the training. To explore the impact of inactivity on response to the intervention, we compared pre-post-retention changes on the WMFT and the JTHF between the subjects who had received physical therapy within a three month period prior to beginning this study (previously active group, N = 6, therapeutic intervention within 3 months) and those who had not had therapy for a longer time (previously inactive group, N = 6, median time post therapeutic intervention = 14 mos.). We evaluated the effects of training on the combined clinical score of the two timed tests (WMFT, JTHF), using a repeated measures ANOVA with a between factor Group (previously active, inactive) and a within factor Measurement time (Pre-test, Post-test, Retention). There was no difference between the two groups (F(1,10) = .06; p = 0.82). Moreover, the Group by Measurement time interaction was not significant (F(2,20) = .260; p = 0.77). These results indicate that the prior level of activity did not affect the outcome of the training.

## Discussion

In this study we tested a rehabilitation paradigm that simultaneously exercised the arm and hand, including the fingers, in an integrated manner using virtual reality task-based gaming simulations. Our goal was to improve hemiparetic hand function in patients in the chronic phase post-stroke. As a group, the subjects were able to more effectively control the upper limb during reaching and hand interaction with the target as demonstrated by improved proximal stability, smoothness and efficiency of the movement path. The improvements in smoothness are indicative of a decrease in the number of sub-movements required to complete the transport phase of the motion. Several authors cite this pattern of change as consistent with improvements in neuromotor control [9,23]. This improved control was in concert with improvement in the distal kinematic measures of fractionation and improved speed. Of note, these changes in robotic measures were accompanied by robust changes in the clinical outcome measures.

Several factors may have influenced the findings in this study. Congruent with the motor learning and neuroplasticity literature, it is believed that the acquisition of a motor skill follows a dose-response relationship [29]. In rehabilitation, the dose is often measured as the number of task repetitions or practice hours. Multiple authors cite the ability of robotically facilitated training to provide highly repetitious training as a key factor for its effectiveness [30,31]. The comparison between the training volume typical to robotic interventions and those of traditional UE interventions is marked. Subjects average over 500 repetitions/day in studies in the robotic rehabilitation literature [32-34] while an observational study of the repetitions performed in a traditional outpatient setting averaged 85 [35]. The average number of repetitions during the two to three hour training sessions used in this study exceeded 2200.

Based upon a review of 20 RCT's, it has been suggested that a minimal dose of at least sixteen hours of practice is required to achieve functional changes [29]. Our subjects performed 22 hours of training, 10 hours during week one and 12 hours in the longer sessions in week two. Each training session in this study was considerably longer than the twenty to ninety minute sessions described in the current robotic literature [30,31] and was delivered within a more concentrated time period [11,34,36-38].

Another factor to consider is that the gaming simulations structured the subjects' attentional focus. It has been shown in people with and without disabilities that the learning of a motor task is more effective when attention is focused on externally rather than on internally based directions [39,40]. In these virtual reality simulations, practice was directed to achieve action goals rather than performing specific movements. The instructions for the game, the feedback provided and the inherent structure of each simulation directed the players' attention to the task to be achieved. In other words, the focus of attention was on the effect of one's movements rather than on the movement itself.

The largest improvements demonstrated with the Virtual Piano were for finger fractionation, which is the ability to flex one finger independently of the other fingers. During practice, the performance feedback, the sound of the appropriate note, occurs when a fractionation target is achieved, reinforcing this construct. In addition fractionation is also specifically reinforced with an adaptive algorithm that increases and decreases the fractionation target, based on the subjects' performance. This algorithm which is described in detail elsewhere appears to help progress the subject towards improved finger function [15]. Subjects made larger improvements in fractionation than speed or accuracy that were not shaped with an algorithm or reinforced with feedback. Similarly, subjects also failed to make improvements in peak finger extension, which was not reinforced with an algorithm, during Hammer Task training. These results are congruent with those of Lum et al. [37] who found that subjects with strokes, training using the MIME system, reduced force direction errors when this construct was shaped with an algorithm.

Day three training performance for the three proximal kinematic measures (hand deviation, path length and trajectory smoothness), deviates from the trend of daily incremental improvement during the rest of the trial (See Figure 3). Three subjects, all with chronic strokes had their worst performance on day three for these measures. This may be secondary to higher levels of fatigue associated with the initiation of an intense training protocol in these subjects. A comparable pattern of high levels of fatigue during the early days of a trial has been demonstrated by a group of CIMT subjects with chronic strokes [41].

Our overarching goal is to integrate development of robotic assisted training devices with the most effective training paradigm for recovery of hand function. It is therefore important to compare the changes in JTHF time in this current study to other studies performed in our lab. In a former study of comparable duration, that trained the hand only, the subjects showed a 10% improvement in the time of the JTHF [6], while in this current study that trained the arm and hand simultaneously, there was a 24 sec decrease in the time to complete the JTHF achieved by the subjects in this study, which was equal to a 20% change in the time needed to complete all the items on the JTHF. This decrease in time represents 27% of the difference between the initial scores of the stroke subjects, and the aged matched controls. Moreover, it represents 33% of the difference between the initials scores of the impaired and uninvolved hand. Given this robust improvement as well as the difference between initial scores for the impaired arm and the less impaired arm, one can suggest that functional changes may have occurred secondary to this training. Future analyses would be required to relate this robust change in the JTHF with changes in activities of daily living function.

Essential factors such as the dosage and intensity of the practice, the focus of attention on the movement outcome, and the drive provided by specific algorithms are important to achieving functional outcomes. However, these factors have been similar in our past studies. What was different in this study was the complexity of the movements required to interact with the virtual simulations. When we trained the hand alone, the gaming simulations were very simple activities, requiring only control of wrist and finger movement. Whereas in this study the activities required by the gaming simulations were more complex and required simultaneous control of integrated shoulder, elbow, forearm, wrist and finger movements. These factors appear to have had a substantial, positive effect on our goal of improving hemiparetic hand function.

However, an important question to consider is whether it is the complexity of the simulations or the consistent training of integrated shoulder, elbow, forearm, wrist and finger movements that is responsible for these improvements. This question engenders another possible training variation. Will the findings be as robust if the subjects train on complex activities that only require independent and discrete upper arm movements or hand movements. To answer this question our lab is in the process of initiating a randomized controlled trial testing for the effect of integrated versus isolated training of proximal and distal upper extremity effectors to compare the outcomes with our previous findings.

## Conclusions

The quasi experimental data presented in this paper lacks the controls necessary to make conclusive statements about the efficacy of this treatment approach. However, double baseline measures indicated that the subjects in this study were neurologically stable. We believe that the magnitude of the changes and the stability of the patient's function prior to training, along with maintenance of several aspects of the gains demonstrated at retention make a compelling argument that this approach to training warrants continued study.

## Competing interests

The authors declare no competing interests with respect to the authorship and/or publication of this article.

## Authors' contributions

All authors read and approved the final manuscript. ASM participated in the robotic/VR system design, study design, data collection, data analysis, initial manuscript preparation and manuscript revision. GGF participated in the study design, subject recruitment, data collection, data analysis, initial manuscript preparation and manuscript revision. QQ participated in the robotic/VR system design, data collection, data analysis and initial manuscript preparation. SS participated in the data collection, manuscript preparation and manuscript revision processes. IL participated in the data collection, manuscript preparation and manuscript revision processes. AD participated in the study design, data analysis and manuscript revision processes. SVA participated in the robotic/VR system design, study design, data collection, data analysis, initial manuscript preparation and manuscript revision processes.
